# Bis{(*E*)-2-eth­oxy-6-[2-(ethyl­ammonio)ethyl­iminometh­yl]phenolato}nickel(II) bis(perchlorate)

**DOI:** 10.1107/S1600536808023684

**Published:** 2008-07-31

**Authors:** Xue-Wen Zhu, Xu-Zhao Yang

**Affiliations:** a Key Laboratory of Surface and Interface Science of Henan, School of Material and Chemical Engineering, Zhengzhou University of Light Industry, Zhengzhou 450002, People’s Republic of China

## Abstract

In the title centrosymmetric mononuclear nickel(II) complex, [Ni(C_13_H_20_N_2_O_2_)_2_](ClO_4_)_2_, the Ni^II^ atom is four-coordinated by the imine N and phenolate O atoms of the zwitterionic forms of two Schiff base ligands in a square-planar coordination geometry. In the crystal structure, mol­ecules are linked through inter­molecular N—H⋯O hydrogen bonds, forming chains running along the *a* axis.

## Related literature

For background to the chemistry of the Schiff base complexes, see: Ali *et al.* (2008 ▶); Biswas *et al.* (2008 ▶); Carlsson *et al.* (2002 ▶, 2004 ▶); Chen *et al.* (2008 ▶); Darensbourg & Frantz (2007 ▶); Habibi *et al.* (2007 ▶); Kawamoto *et al.* (2008 ▶); Tomat *et al.* (2007 ▶); Wu *et al.* (2008 ▶); Yuan *et al.* (2007 ▶). For related structures, see: Ma *et al.* (2008 ▶); Skovsgaard *et al.* (2005 ▶); Zhao (2007 ▶).
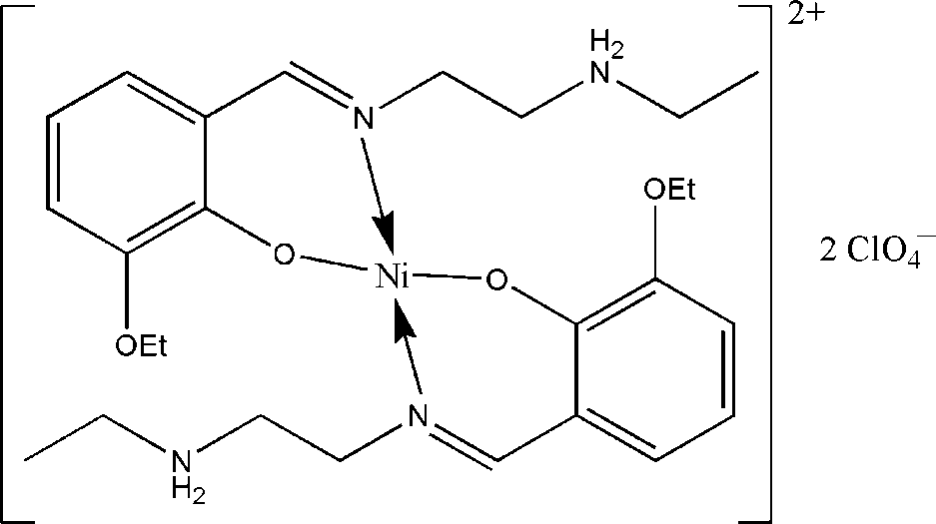


## Experimental

### 

#### Crystal data

[Ni(C_13_H_20_N_2_O_2_)_2_](ClO_4_)_2_
*M* *_r_* = 730.23
Monoclinic, 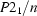

*a* = 8.386 (3) Å
*b* = 8.566 (3) Å
*c* = 21.862 (6) Å
β = 99.068 (4)°
*V* = 1550.8 (9) Å^3^
*Z* = 2
Mo *K*α radiation
μ = 0.87 mm^−1^
*T* = 298 (2) K
0.23 × 0.20 × 0.20 mm

#### Data collection

Bruker APEXII CCD area-detector diffractometer
Absorption correction: multi-scan (*SADABS*; Sheldrick, 2004 ▶) *T*_min_ = 0.826, *T*_max_ = 0.846
12509 measured reflections
3363 independent reflections
2770 reflections with *I* > 2σ(*I*)
*R*_int_ = 0.041

#### Refinement

*R*[*F* ^2^ > 2σ(*F* ^2^)] = 0.039
*wR*(*F* ^2^) = 0.102
*S* = 1.04
3363 reflections
207 parameters
H-atom parameters constrained
Δρ_max_ = 0.28 e Å^−3^
Δρ_min_ = −0.34 e Å^−3^

### 

Data collection: *APEX2* (Bruker, 2004 ▶); cell refinement: *SAINT* (Bruker, 2004 ▶); data reduction: *SAINT*; program(s) used to solve structure: *SHELXS97* (Sheldrick, 2008 ▶); program(s) used to refine structure: *SHELXL97* (Sheldrick, 2008 ▶); molecular graphics: *SHELXTL* (Sheldrick, 2008 ▶); software used to prepare material for publication: *SHELXTL*.

## Supplementary Material

Crystal structure: contains datablocks global, I. DOI: 10.1107/S1600536808023684/sj2526sup1.cif

Structure factors: contains datablocks I. DOI: 10.1107/S1600536808023684/sj2526Isup2.hkl

Additional supplementary materials:  crystallographic information; 3D view; checkCIF report

## Figures and Tables

**Table:** 

Ni1—O1	1.836 (2)
Ni1—N1	1.910 (2)

**Table:** 

O1^i^—Ni1—O1	180
O1^i^—Ni1—N1	87.67 (7)
O1—Ni1—N1	92.33 (7)
N1^i^—Ni1—N1	180

Symmetry code: (i) 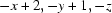.

**Table 2:** Hydrogen-bond geometry (Å, °)

*D*—H⋯*A*	*D*—H	H⋯*A*	*D*⋯*A*	*D*—H⋯*A*
N2—H2*B*⋯O2^i^	0.90	2.34	3.013 (3)	131
N2—H2*B*⋯O1^i^	0.90	1.97	2.764 (2)	146
N2—H2*A*⋯O3	0.90	2.56	3.242 (3)	132
N2—H2*A*⋯O3^ii^	0.90	2.13	2.916 (3)	145

Symmetry codes: (i) 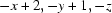; (ii) 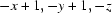.

**Fig. 1.**
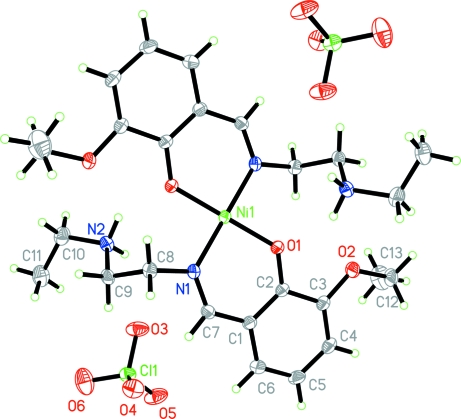
The molecular structure of (I) with ellipsoids drawn at the 30% probability level. Unlabelled atoms are at the symmetry positions 2 - x, 1 - y, - z.

**Fig. 2.**
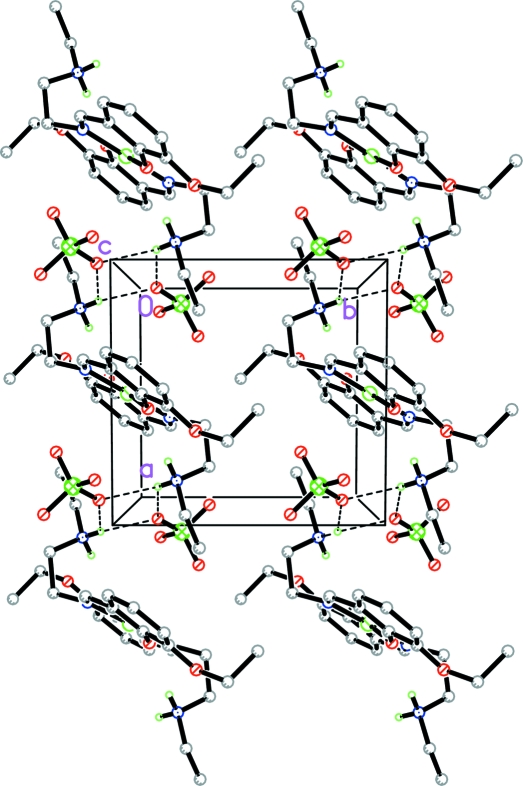
The crystal packing of (I), viewed along the c axis.

## supplementary crystallographic information

### Comment

Schiff bases have widely been used as versatile ligands in coordination chemistry (Biswas *et al.*, 2008; Wu *et al.*, 2008; Kawamoto *et al.*, 2008; Ali *et al.*, 2008; Habibi *et al.*, 2007), and their metal complexes are of great interest in many fields (Chen *et al.*, 2008; Yuan *et al.*, 2007; Tomat *et al.*, 2007; Darensbourg & Frantz, 2007). Nickel(II) is present in the active sites of urease (Carlsson *et al.*, 2002, 2004). In this paper, a new nickel(II) complex, (I), Fig. 1, with the Schiff base ligand (E)-2-ethoxy-6-((3-(methylamino)propylimino)methyl)phenol has been synthesized and structurally characterized.

Complex (I) consists of a centrosymmetric mononuclear nickel(II) complex cation and two perchlorate anions. The Ni^II^ atom in the cation, lies on an inversion centre, with the asymmetric unit made up from one half of the Ni(II) complex and one perchlorate anion.  The Ni(II) atom  is four-coordinated by two imine N and two phenolate O atoms from two zwitterionic Schiff base ligands in a square-planar coordination geometry. The coordinate bond lengths (Table 1) are typical and comparable to the corresponding values observed in similar nickel(II) Schiff base complexes (Zhao, 2007; Skovsgaard *et al.*, 2005; Ma *et al.*, 2008).

In the crystal structure, molecules are linked through intermolecular N–H···O hydrogen bonds (Table 2), forming chains running along the *a* axis (Fig. 2).

### Experimental

The Schiff base compound was prepared by the condensation of equimolar amounts of 3-ethoxysalicylaldehyde with *N*-ethylethane-1,2-diamine in a methanol solution. The complex was prepared by the following method. To a methanol solution (5 ml) of Ni(ClO_4_)_2_.6H_2_O (36.6 mg, 0.1 mmol) was added a methanol solution (10 ml) of the Schiff base compound (23.6 mg, 0.1 mmol) with stirring. The mixture was stirred for 30 min at room temperature and filtered. Upon keeping the filtrate in air for a few days, red block-shaped crystals formed at the bottom of the vessel on slow evaporation of the solvent.

### Refinement

All H atoms were placed in geometrically idealized positions and constrained to ride on their parent atoms, with C–H distances in the range 0.93–0.97 Å, N–H distances of 0.90 Å, and with *U*_iso_(H) = 1.2*U*_eq_(C,*N*) and 1.5*U*_eq_(methyl C).

### Crystal data

**Table:**

[Ni(C_13_H_20_N_2_O_2_)_2_](ClO_4_)_2_	*F*_000_ = 764
*M**_r_* = 730.23	*D*_x_ = 1.564 Mg m^−^^3^
Monoclinic, *P*2_1_/*n*	Mo *K*α radiation λ = 0.71073 Å
Hall symbol: -P 2yn	Cell parameters from 3252 reflections
*a* = 8.386 (3) Å	θ = 2.5–25.4º
*b* = 8.566 (3) Å	µ = 0.87 mm^−^^1^
*c* = 21.862 (6) Å	*T* = 298 (2) K
β = 99.068 (4)º	Block, red
*V* = 1550.8 (9) Å^3^	0.23 × 0.20 × 0.20 mm
*Z* = 2	

### Data collection

**Table:**

Bruker APEXII CCD area-detector diffractometer	3363 independent reflections
Radiation source: fine-focus sealed tube	2770 reflections with *I* > 2σ(*I*)
Monochromator: graphite	*R*_int_ = 0.041
*T* = 298(2) K	θ_max_ = 27.0º
ω scans	θ_min_ = 1.9º
Absorption correction: multi-scan(SADABS; Sheldrick, 2004)	*h* = −10→10
*T*_min_ = 0.826, *T*_max_ = 0.846	*k* = −10→10
12509 measured reflections	*l* = −27→27

### Refinement

**Table:**

Refinement on *F*^2^	Secondary atom site location: difference Fourier map
Least-squares matrix: full	Hydrogen site location: inferred from neighbouring sites
*R*[*F*^2^ > 2σ(*F*^2^)] = 0.039	H-atom parameters constrained
*wR*(*F*^2^) = 0.102	*w* = 1/[σ^2^(*F*_o_^2^) + (0.0512*P*)^2^ + 0.4599*P*] where *P* = (*F*_o_^2^ + 2*F*_c_^2^)/3
*S* = 1.04	(Δ/σ)_max_ = 0.001
3363 reflections	Δρ_max_ = 0.28 e Å^−^^3^
207 parameters	Δρ_min_ = −0.34 e Å^−^^3^
Primary atom site location: structure-invariant direct methods	Extinction correction: none

### Special details

**Table:**

Geometry. All e.s.d.'s (except the e.s.d. in the dihedral angle between two l.s. planes) are estimated using the full covariance matrix. The cell e.s.d.'s are taken into account individually in the estimation of e.s.d.'s in distances, angles and torsion angles; correlations between e.s.d.'s in cell parameters are only used when they are defined by crystal symmetry. An approximate (isotropic) treatment of cell e.s.d.'s is used for estimating e.s.d.'s involving l.s. planes.
Refinement. Refinement of *F*^2^ against ALL reflections. The weighted *R*-factor *wR* and goodness of fit *S* are based on *F*^2^, conventional *R*-factors *R* are based on *F*, with *F* set to zero for negative *F*^2^. The threshold expression of *F*^2^ > σ(*F*^2^) is used only for calculating *R*-factors(gt) *etc*. and is not relevant to the choice of reflections for refinement. *R*-factors based on *F*^2^ are statistically about twice as large as those based on *F*, and *R*- factors based on ALL data will be even larger.

### Fractional atomic coordinates and isotropic or equivalent isotropic displacement parameters (Å^2^)

**Table:**

	*x*	*y*	*z*	*U*_iso_*/*U*_eq_	
Ni1	1.0000	0.5000	0.0000	0.02592 (13)	
Cl1	0.39043 (7)	0.71469 (7)	0.09059 (3)	0.03882 (17)	
O1	1.06353 (18)	0.41002 (18)	0.07614 (7)	0.0333 (4)	
O2	1.1550 (2)	0.2125 (2)	0.16406 (8)	0.0451 (4)	
O3	0.4539 (3)	0.6169 (2)	0.04665 (9)	0.0624 (6)	
O4	0.5075 (2)	0.8315 (2)	0.11174 (9)	0.0547 (5)	
O5	0.3594 (3)	0.6194 (2)	0.14062 (9)	0.0652 (6)	
O6	0.2480 (3)	0.7874 (3)	0.06108 (13)	0.0790 (7)	
N1	0.8973 (2)	0.6697 (2)	0.03503 (8)	0.0272 (4)	
N2	0.6314 (2)	0.7051 (2)	−0.07021 (9)	0.0342 (4)	
H2A	0.6031	0.6259	−0.0471	0.041*	
H2B	0.7139	0.6718	−0.0886	0.041*	
C1	0.8960 (3)	0.5512 (3)	0.13583 (10)	0.0307 (5)	
C2	1.0022 (3)	0.4303 (3)	0.12705 (9)	0.0281 (5)	
C3	1.0473 (3)	0.3232 (3)	0.17637 (10)	0.0322 (5)	
C4	0.9828 (3)	0.3361 (3)	0.22998 (10)	0.0385 (6)	
H4	1.0109	0.2640	0.2616	0.046*	
C5	0.8759 (3)	0.4560 (3)	0.23756 (11)	0.0436 (6)	
H5	0.8327	0.4634	0.2741	0.052*	
C6	0.8344 (3)	0.5623 (3)	0.19183 (11)	0.0399 (6)	
H6	0.7644	0.6433	0.1976	0.048*	
C7	0.8602 (3)	0.6692 (3)	0.09004 (10)	0.0308 (5)	
H7	0.8036	0.7555	0.1010	0.037*	
C8	0.8632 (3)	0.8182 (2)	0.00079 (11)	0.0317 (5)	
H8A	0.9308	0.8242	−0.0313	0.038*	
H8B	0.8923	0.9044	0.0291	0.038*	
C9	0.6887 (3)	0.8363 (3)	−0.02871 (11)	0.0333 (5)	
H9A	0.6219	0.8436	0.0036	0.040*	
H9B	0.6765	0.9328	−0.0522	0.040*	
C10	0.4917 (3)	0.7422 (3)	−0.11922 (13)	0.0487 (7)	
H10A	0.5245	0.8215	−0.1464	0.058*	
H10B	0.4629	0.6493	−0.1439	0.058*	
C11	0.3475 (3)	0.7982 (4)	−0.09446 (14)	0.0568 (8)	
H11A	0.3152	0.7211	−0.0670	0.085*	
H11B	0.2610	0.8158	−0.1281	0.085*	
H11C	0.3728	0.8940	−0.0723	0.085*	
C12	1.1937 (3)	0.0850 (3)	0.20663 (12)	0.0465 (6)	
H12A	1.2110	0.1255	0.2486	0.056*	
H12B	1.2937	0.0372	0.1992	0.056*	
C13	1.0661 (5)	−0.0355 (4)	0.2011 (2)	0.0790 (11)	
H13A	0.9690	0.0091	0.2116	0.119*	
H13B	1.1007	−0.1201	0.2288	0.119*	
H13C	1.0458	−0.0737	0.1593	0.119*	

### Atomic displacement parameters (Å^2^)

**Table:**

	*U*^11^	*U*^22^	*U*^33^	*U*^12^	*U*^13^	*U*^23^
Ni1	0.0286 (2)	0.0257 (2)	0.0236 (2)	0.00323 (15)	0.00463 (15)	0.00429 (15)
Cl1	0.0470 (4)	0.0310 (3)	0.0392 (3)	−0.0058 (2)	0.0091 (3)	−0.0041 (2)
O1	0.0407 (9)	0.0352 (9)	0.0255 (7)	0.0112 (7)	0.0096 (7)	0.0078 (6)
O2	0.0555 (11)	0.0445 (10)	0.0376 (9)	0.0190 (8)	0.0145 (8)	0.0175 (8)
O3	0.1038 (17)	0.0375 (10)	0.0533 (12)	−0.0086 (11)	0.0357 (11)	−0.0095 (9)
O4	0.0541 (12)	0.0456 (11)	0.0639 (13)	−0.0157 (9)	0.0075 (9)	−0.0132 (9)
O5	0.1053 (17)	0.0525 (12)	0.0438 (11)	−0.0208 (12)	0.0300 (11)	−0.0021 (9)
O6	0.0493 (13)	0.0656 (14)	0.116 (2)	−0.0016 (11)	−0.0058 (12)	0.0108 (14)
N1	0.0264 (9)	0.0250 (9)	0.0292 (9)	−0.0002 (7)	0.0015 (7)	0.0020 (7)
N2	0.0319 (10)	0.0294 (10)	0.0408 (11)	0.0026 (8)	0.0038 (8)	−0.0007 (8)
C1	0.0301 (11)	0.0345 (12)	0.0277 (11)	−0.0006 (9)	0.0048 (9)	−0.0003 (9)
C2	0.0290 (11)	0.0310 (11)	0.0244 (10)	−0.0028 (9)	0.0043 (9)	0.0013 (9)
C3	0.0323 (12)	0.0354 (12)	0.0284 (11)	−0.0017 (10)	0.0030 (9)	0.0047 (9)
C4	0.0413 (14)	0.0464 (14)	0.0271 (11)	−0.0036 (11)	0.0034 (10)	0.0079 (10)
C5	0.0465 (15)	0.0580 (16)	0.0288 (12)	0.0011 (13)	0.0132 (11)	0.0006 (11)
C6	0.0391 (13)	0.0466 (14)	0.0356 (13)	0.0061 (11)	0.0107 (10)	−0.0031 (11)
C7	0.0292 (11)	0.0299 (12)	0.0333 (12)	0.0024 (9)	0.0048 (9)	−0.0033 (9)
C8	0.0359 (12)	0.0224 (11)	0.0362 (12)	−0.0029 (9)	0.0033 (10)	0.0010 (9)
C9	0.0374 (13)	0.0232 (11)	0.0387 (12)	0.0043 (9)	0.0042 (10)	0.0019 (9)
C10	0.0474 (15)	0.0519 (16)	0.0429 (14)	−0.0002 (13)	−0.0052 (12)	0.0011 (12)
C11	0.0354 (14)	0.0620 (19)	0.068 (2)	−0.0005 (13)	−0.0059 (13)	0.0023 (15)
C12	0.0508 (16)	0.0441 (15)	0.0445 (14)	0.0123 (12)	0.0072 (12)	0.0191 (12)
C13	0.078 (2)	0.0510 (19)	0.107 (3)	−0.0058 (18)	0.011 (2)	0.010 (2)

### Geometric parameters (Å, °)

**Table:**

Ni1—O1^i^	1.836 (2)	C4—H4	0.9300
Ni1—O1	1.836 (2)	C5—C6	1.357 (4)
Ni1—N1^i^	1.910 (2)	C5—H5	0.9300
Ni1—N1	1.910 (2)	C6—H6	0.9300
Cl1—O6	1.410 (2)	C7—H7	0.9300
Cl1—O5	1.421 (2)	C8—C9	1.512 (3)
Cl1—O4	1.4268 (18)	C8—H8A	0.9700
Cl1—O3	1.4384 (19)	C8—H8B	0.9700
O1—C2	1.309 (2)	C9—H9A	0.9700
O2—C3	1.365 (3)	C9—H9B	0.9700
O2—C12	1.439 (3)	C10—C11	1.481 (4)
N1—C7	1.289 (3)	C10—H10A	0.9700
N1—C8	1.481 (3)	C10—H10B	0.9700
N2—C9	1.476 (3)	C11—H11A	0.9600
N2—C10	1.492 (3)	C11—H11B	0.9600
N2—H2A	0.9000	C11—H11C	0.9600
N2—H2B	0.9000	C12—C13	1.478 (4)
C1—C2	1.399 (3)	C12—H12A	0.9700
C1—C6	1.405 (3)	C12—H12B	0.9700
C1—C7	1.421 (3)	C13—H13A	0.9600
C2—C3	1.421 (3)	C13—H13B	0.9600
C3—C4	1.370 (3)	C13—H13C	0.9600
C4—C5	1.390 (4)		
			
O1^i^—Ni1—O1	180.0	C1—C6—H6	119.7
O1^i^—Ni1—N1^i^	92.33 (7)	N1—C7—C1	127.2 (2)
O1—Ni1—N1^i^	87.67 (7)	N1—C7—H7	116.4
O1^i^—Ni1—N1	87.67 (7)	C1—C7—H7	116.4
O1—Ni1—N1	92.33 (7)	N1—C8—C9	113.63 (18)
N1^i^—Ni1—N1	180.0	N1—C8—H8A	108.8
O6—Cl1—O5	111.19 (15)	C9—C8—H8A	108.8
O6—Cl1—O4	109.16 (13)	N1—C8—H8B	108.8
O5—Cl1—O4	110.69 (13)	C9—C8—H8B	108.8
O6—Cl1—O3	109.13 (15)	H8A—C8—H8B	107.7
O5—Cl1—O3	108.15 (12)	N2—C9—C8	112.54 (18)
O4—Cl1—O3	108.46 (13)	N2—C9—H9A	109.1
C2—O1—Ni1	128.20 (14)	C8—C9—H9A	109.1
C3—O2—C12	119.27 (19)	N2—C9—H9B	109.1
C7—N1—C8	114.76 (18)	C8—C9—H9B	109.1
C7—N1—Ni1	124.19 (15)	H9A—C9—H9B	107.8
C8—N1—Ni1	120.95 (14)	C11—C10—N2	113.6 (2)
C9—N2—C10	114.96 (19)	C11—C10—H10A	108.8
C9—N2—H2A	108.5	N2—C10—H10A	108.8
C10—N2—H2A	108.5	C11—C10—H10B	108.8
C9—N2—H2B	108.5	N2—C10—H10B	108.8
C10—N2—H2B	108.5	H10A—C10—H10B	107.7
H2A—N2—H2B	107.5	C10—C11—H11A	109.5
C2—C1—C6	119.9 (2)	C10—C11—H11B	109.5
C2—C1—C7	119.90 (19)	H11A—C11—H11B	109.5
C6—C1—C7	120.0 (2)	C10—C11—H11C	109.5
O1—C2—C1	123.96 (19)	H11A—C11—H11C	109.5
O1—C2—C3	117.8 (2)	H11B—C11—H11C	109.5
C1—C2—C3	118.25 (19)	O2—C12—C13	113.0 (2)
O2—C3—C4	126.0 (2)	O2—C12—H12A	109.0
O2—C3—C2	113.84 (19)	C13—C12—H12A	109.0
C4—C3—C2	120.2 (2)	O2—C12—H12B	109.0
C3—C4—C5	120.7 (2)	C13—C12—H12B	109.0
C3—C4—H4	119.7	H12A—C12—H12B	107.8
C5—C4—H4	119.7	C12—C13—H13A	109.5
C6—C5—C4	120.2 (2)	C12—C13—H13B	109.5
C6—C5—H5	119.9	H13A—C13—H13B	109.5
C4—C5—H5	119.9	C12—C13—H13C	109.5
C5—C6—C1	120.7 (2)	H13A—C13—H13C	109.5
C5—C6—H6	119.7	H13B—C13—H13C	109.5

Symmetry codes: (i) −*x*+2, −*y*+1, −*z*.

### Hydrogen-bond geometry (Å, °)

**Table:**

*D*—H···*A*	*D*—H	H···*A*	*D*···*A*	*D*—H···*A*
N2—H2B···O2^i^	0.90	2.34	3.013 (3)	131
N2—H2B···O1^i^	0.90	1.97	2.764 (2)	146
N2—H2A···O3	0.90	2.56	3.242 (3)	132
N2—H2A···O3^ii^	0.90	2.13	2.916 (3)	145

Symmetry codes: (i) −*x*+2, −*y*+1, −*z*; (ii) −*x*+1, −*y*+1, −*z*.
